# Prophylactic Lamivudine to Improve the Outcome of Breast Cancer Patients With HBsAg Positive During Chemotherapy: A Meta-Analysis

**DOI:** 10.5812/hepatmon.6496

**Published:** 2013-04-01

**Authors:** Yihu Zheng, Shengchu Zhang, Hooi Min Tan Grahn, Chao Ye, Zheng Gong, Qiyu Zhang

**Affiliations:** 1 Department of General Surgery, The First Affiliated Hospital, Wenzhou Medical College, Wenzhou, Zhejiang, China; 2 Department of General Surgery, Yichang Central People’s Hospital, The First Clinical Medical College of Three Gorges University, Yichang, China; 3 Metabolism, Obesity/Diabetes, Department of Biochemistry, Boston University School of Medicine, Boston, USA; 4 State Key Laboratory for Diagnosis and Treatment of Infectious Disease, The First Affiliated Hospital, Zhejiang University, Hangzhou, China

**Keywords:** Breast Neoplasms, Drug Therapy, Hepatitis B Virus, Lamivudine, Meta-Analysis, Drug Toxicity, Preventive Medicin

## Abstract

**Context:**

Raising the chemotherapy-induced HBV reactivation is parallel to the increment of chemotherapy treatments in breast cancer patients. This meta-analysis aims to evaluate the efficacy of prophylactic use of lamivudine in breast cancer patients with HBsAg positive during chemotherapy.

**Evidence Acquisition:**

MEDLINE, Pubmed, Ovid and Embase were used to search for clinical studies comparing with or without prophylactic use of lamivudine for HBV reactivation in breast cancer patients receiving chemotherapy. Outcomes of interest were the rate of HBV reactivation, incidence of hepatitis and incidence of hepatitis attributable to HBV reactivation, severity of hepatitis and severity of hepatitis attributable to HBV reactivation, the rate of chemotherapy disruption, and the rate of chemotherapy disruption attributable to HBV reactivation, overall mortality, and mortality attributable to HBV reactivation.

**Results:**

Four studies with 285 patients were included in this meta-analysis. The rate of HBV reactivation, incidence of hepatitis and incidence of hepatitis related to HBV reactivation were reduced by use of prophylactic lamivudine compared to control group. Pooled Odds Ratios (ORs) were 0.09 (95% confidence intervals [CI] 0.03-0.26; P < 0.0001), 0.23 (95% CI 0.06-0.92; P = 0.04), and 0.10 (95% CI 0.03-0.32; P < 0.0001) respectively. There was a reduction in chemotherapy disruption related to HBV reactivation by use of prophylactic lamivudine (pooled OR = 0.11; 95% CI 0.02-0.58; P = 0.01). Chemotherapy disruption, overall mortality, and mortality attributable to HBV reactivation were not significantly different between two groups. Pooled ORs were 0.42 (95% CI 0.11-1.58; P = 0.20), 0.37 (95% CI 0.07-2.04; P = 0.25), and 0.25 (95% CI 0.01-6.82; P = 0.41) respectively. Lamivudine was well-tolerated, and no additional toxicity was observed.

**Conclusions:**

Use of prophylactic lamivudine may have positive effect on the outcome of breast cancer patients with HBsAg positive during chemotherapy.

## 1. Context

Hepatitis B virus (HBV) reactivation is a well-known complication of cytotoxic chemotherapy for malignancy (1, 2). The reports of HBV reactivation of patients with hematologic malignancies has been gained recently (3-8), but there are reports concerning reactivation in patients with solid tumors (9-11). In patients with solid tumors receiving chemotherapy, the highest rates of HBV reactivation have been reported in breast cancer patients and the incidence ranges are between 41% and 56% (12, 13). There is a great diversity of clinical presentation while HBV reactivation, ranging from a subclinical and asymptomatic elevation of hepatic enzymes to severe acute hepatitis and even death resulting from fulminant hepatic failure is reported (14). Moreover, delaying or premature termination of chemotherapy may also compromise these patients’ prognosis (13).

Lamivudine, a nucleoside analogue, has a beneficial effect on preventing HBV reactivation and HBV-related death in patients with HBV surface of positive antigen (HBsAg) e undergoing chemotherapy (15-17). Most relevant studies focused on patients with lymphoma (18-20), whereas information on breast cancer patients has been missed (21-23). Further, the application of prophylactic lamivudine for HBV reactivation in chemotherapy remains controversial and is not standardized (24). We conducted a meta-analysis to assess the efficacy of use of prophylactic lamvudine on preventing HBV reactivation, hepatitis, severity of hepatitis, disruption of chemotherapy and mortality in breast cancer patients with HBsAg positive receiving systemic chemotherapy.

## 2. Evidence Acquisition

### 2.1. Search Methods for the Identification of Studies

The electronic databases such as MEDLINE, Pubmed, Ovid and Embase were used to search all clinical studies with or without prophylactic use of lamivudinec for hepatitis B reactivation in breast cancer patients receiving chemotherapy. The literature searches were carried out using following medical subject headings (MeSH) and free text words: “lamivudine”, “chemotherapy”, “cancer”, “carcinoma”, “neoplasm”, “malignant” and “breast”. We also checked the reference lists of all identified studies If multiple trials were derived from the same or partly overlapping study populations, only the largest or most recent eligible trial with detailed information would be included. The searches of the entire databases were conducted by June 2011. No language and time restrictions were considered in the course of articles searching.

### 2.2. Outcomes of Interest and Definitions

The primary outcomes were the rate of HBV reactivation, incidence of hepatitis and incidence of hepatitis attributable to HBV reactivation, rate of chemotherapy disruption, and rate of chemotherapy disruption attributable to HBV reactivation and overall mortality and mortality attributable to HBV reactivation. The secondary outcomes were severity of hepatitis and severity of hepatitis attributable to HBV reactivation. According to the definition initially described by Lok et al. (5) and subsequently modified by Yeo et al. (10), hepatitis was defined as more than three times increase in alanine aminotransferase (ALT) that exceeded the upper limit of normal range (ULN) or an absolute increase of ALT which is more than 100 U/L compared to baseline pre-chemotherapy value. The ULN was different based on different levels of individual studies. Hepatitis attributable to HBV reactivation was defined as an increase in HBV DNA levels of more than 10 times compared to the baseline level or an absolute increase of HBV DNA levels that exceeded 1 × 109 copies/ml, in the absence of other systemic infection. The severity of hepatitis was defined as ‘mild’, ‘moderate’, and ‘severe’ when the rise in ALT was ≤ 2 × ULN, > 2 × and ≤ 5 × ULN, and > 5 × UNL respectively. Chemotherapy disruption was defined as either a premature termination of chemotherapy or a delay of more than 8 days of chemotherapy between cycles. The death related to HBV reactivation was defined as death of a patient who had documented HBV reactivation that was reported to be as an HBV-related death and had no other apparent cause of death.

### 2.3. Inclusion and Exclusion Criteria

The studies in this meta-analysis included two arms of prophylactic use of lamivudine (the prophylactic lamivudine group) and non-prophylactic use of lamivudine (the control group) to prevent HBV reactivation in breast cancer patients with HBsAg positive during systemic chemotherapy, irrespective of either randomized, controlled studies, or retrospective and prospective cohort studies. Studies were not done if no relevant data could be extracted. Patients who had been treated with anti-HBV therapy within the previous 6 months were excluded. Patients with HIV co-infection were excluded, too. The study with the largest number of patients and explicit details was selected among reduplicative studies.

### 2.4. Study Selection and Date Extraction

Two reviewers independently screened titles and abstracts for inclusion and exclusion according to the inclusion criteria and the exclusion criteria. Data were extracted from selected study including demographic data, baseline ALT, viral marker status [HBsAg, anti-HBV surface antibody (HBsAb), HBV envelope antigen (HBeAg), anti-HBV envelope antibody (HBeAb), HBV core antigen (HBcAg), anti-HBV core antibody (HBcAb) and HBV DNA quantitation], rate of HBV reactivation, incidence of hepatitis, severity of hepatitis, chemotherapy disruption, overall mortality, incidence of hepatitis and severity of hepatitis attributable to HBV reactivation, chemotherapy disruption and mortality attributable to HBV reactivation. Any disagreements between reviewers will be resolved by consensus or if necessary by arbitration done by a third reviewer. For each data which were not clear or not presented by the authors in the publications, we attempted to contact the authors for more details.

### 2.5. Statistical Analysis

All interesting outcomes were dichotomous data and were presented as an odds ratio (OR) with 95% confidence intervals (CI). Statistical homogeneity of effects was evaluated using chi-square (Chi2) and I-square (13) tests, with P < 0.1 indicating significant heterogeneity. There was obvious clinical heterogeneity due to variant chemotherapeutics in each study or even in each group. So random effect model was used to estimate the pooling effect of outcomes even in the situation where no significant heterogeneity was confirmed. Sensitive analysis was carried out by excluding the heterogeneity study or the study of the least sample size depending on the presence or absence of significant heterogeneity. Potential publication bias in the meta-analysis was assessed by Begg's Test. Statistical significance was analyzed by P value (P < 0.05). The Cochrane Collaboration’s Review Manager Software (RevMan version 5.0; Oxford, United Kingdom) was used for data analysis, and the Stata version 10 (Computer Resource Center, Atlanta, Ameriman) was used for the assessment of potential publication bias.

## 3. Results

**Figure 1.**
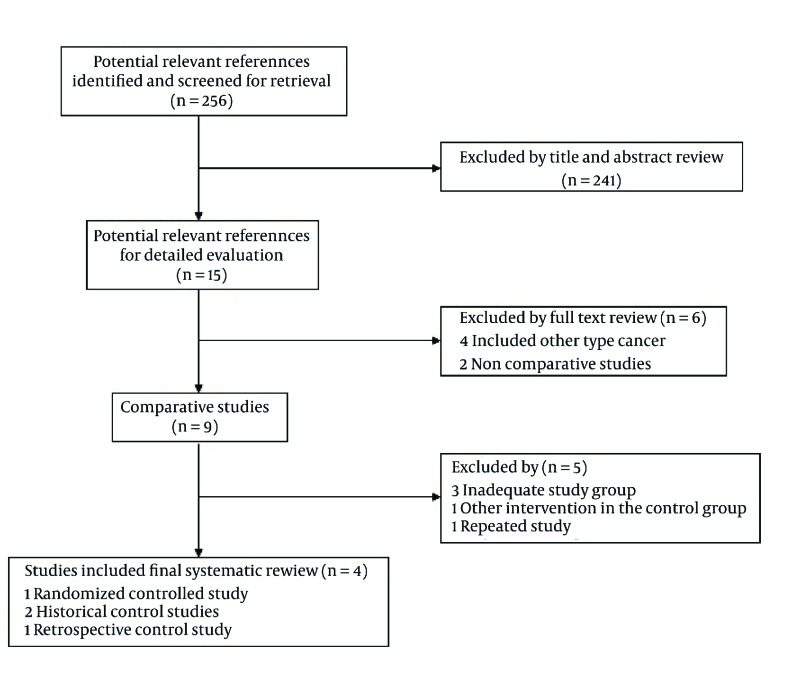
Modified Flow Chart According to the QUOROM Statement Summarizing the Number of Screened Abstracts and Identified Relevant Articles During the Review Process

All of the 256 studies were identified. By scanning titles and abstracts, 241 redundant publications, trial, and review were excluded. After referring to full texts, 6 studies that did not fulfill the inclusion criteria were removed. Five studies were excluded from the remaining nine comparative studies. The flow diagram of the trial selection process was shown in Figure 1.

Four studies with 285 patients were included in this study (12, 23, 25, 26). One study was the prospective randomized controlled study (25), the other two studies were longitudinal historic controlled studies (12) and the remaining one is retrospective controlled study (26). The baseline characteristics of the four included studies were summarized in Table 1. All of the patients of four studies were from East Asia, three (12, 23, 25) from China and one (26) from Korea. The four studies (12, 23, 25, 26) concentrated on breast cancer patients with HBsAg positive and only two (12, 25) provided the baseline HBV, DNA prior to chemotherapy. There were no significant differences among baseline study characteristics with regards to patients’ age and baseline ALT prior to chemotherapy between the prophylactic and the control group in four studies. Chemotherapeutic regimens were not significantly different in three studies (12, 25, 26), but a higher proportion of anthracycline-based chemotherapeutic regimens in the prophylactic group were found compared to the control group in one study (23). The interesting outcomes included in the meta-analysis were shown in Table 2.

**Table 1.** The Baseline Characteristics of the Four Trials

	Dai et al. (2004)		Yeo et al. (2004)		Long et al. (2011)		Yun et al. (2011)	
	P	C	P	C	P	C	P	C
**No. of Patients**	11	9	31	61	21	21	55	76
**Gender, female/male**	NM	NM	31/0	61/0	21/0	21/0	55/0	76/0
**Age, y, Median (Range)**	47 (36-58)	43 (27-55)	46 (31-68)	46 (31-71)	45 (29-64)	43 (20-62)	48 (30-68)	46 (30-69)
**Baseline ALT Median (Range), IU/l**	14 (12-31)	15 (6-54)	28 (13-137)	27 (10-98)	22.3 (7.0-96.0)	14.6 (6.0-27.0)	25 a	25 a
**Baseline Viral Status**								
HBsAg, +/-	11/0	9/0	31/0	61/0	21/0	21/0	55/0	76/0
HBsAb, +/-	NM	NM	NM	NM	2/19	1/20	NM	NM
HBeAg, +/-	2/9	1/8	NM	NM	2/19	3/18	NM	NM
HBeAb, +/-	9/2	8/1	NM	NM	18/3	17/4	NM	NM
HBcAg, +/-/missing	NM	NM	NM	NM	2/14/5	3/13/5	NM	NM
HBcAb, +/-	NM	NM	NM	NM	21/0	20/1	NM	NM
HBV-DNA, log copies/ml	3.11 a	2.57 a	NM	NM	6.16×10 ^6^ b	3.99×10 ^6^ b	NM	NM
**Chemotherapy Regimen**								
Anthracyline Based	5	4	30	36	2	1	28	45
Taxane Based	0	2	NM	NM	7	4	0	0
Anthracyline and Taxane Based	5	3	NM	NM	10	16	27	31
Others	1	0	NM	NM	2	0	0	0
**Type of Trial**	Randomized controlled study		Historic controlled study		Historic controlled study		Retrospective controlled study	

Abbreviations: ALT, alanine aminotransferase; C, the control group; NM, non-mentioned; P, the prophylactic lamivudine group

^a^Mean

^b^Median

**Table 2.** The Results for Various Outcomes of the Four Trials

	Dai et al. (2004)		Yeo et al. (2004)		Long et al. (2011)		Yun et al. (2011)	
	P	C	P	C	P	C	P	C
**HBV reactivation**	0	5	2	19	0	6	1	16
**Hepatitis**	0	5	4	36	5	3	5	25
**Hepatitis Attributable to HBV Reactivation**	0	4	2	19	0	0	1	16
**Severity of hepatitis**								
Mild	1	0	1	11	3	2	3	3
Moderate	2	1	0	13	0	1	2	7
Severe	0	4	3	12	2	0	0	15
**Severity of Hepatitis Attributable to HBV Reactivation**								
Mild	0	0	0	8	0	0	0	2
Moderate	0	1	0	7	0	0	1	1
Severe	0	3	2	4	0	0	0	13
**Chemotherapy Disruption**	NM	NM	5	28	4	2	2	11
**Chemotherapy Disruption Attributable to HBV Reactivation**	NM	NM	1	13	0	0	0	7
**Overall Mortality**	1	2	NM	NM	0	1	0	1
**Mortality Attributable to HBV Reactivation**	0	1	NM	NM	0	0	0	0

Abbreviations: C, the control group; NM, non-mentioned; P, the prophylactic lamivudine group We made a mistake in Abbreviations.

### 3.1. Primary Outcome

There were significant differences in counterpart groups in various outcomes, including rate of HBV reactivation [2.5% vs. 27.5% pooled OR = 0.09, 95%CI (0.03, 0.26), P < 0.0001] (Table 3), incidence of hepatitis [11.9% vs. 41.3%, pooled OR = 0.23, 95%CI (0.06, 0.92), P = 0.04] (Table 3), incidence of hepatitis attributable to HBV reactivation [2.5% vs. 23.5%, pooled OR = 0.10, 95%CI (0.03, 0.32), P < 0.0001] (Table 3) and rate of chemotherapy disruption attributable to HBV reactivation [0.01% vs. 12.7%, pooled OR = 0.11, 95%CI (0.02, 0.58), P = 0.01] (Table 3). It is suggested that the outcomes were in favor of the prophylactic lamivudine group. Heterogeneity was not found in the rate of HBV reactivation (P = 0.81, Table 3), incidence of hepatitis attributable to HBV reactivation (P = 0.75, Table 3) and the rate of chemotherapy disruption attributable to HBV reactivation (P = 0.83, Table 3). However, it exhibited significant heterogeneity in incidence of hepatitis (P = 0.02, Table 3) which could be due to the trial of Long et al. (25). Sensitive analysis showed that there was still significant difference in this four outcome measures (Table 3).

Comparison between the prophylactic lamivudine and the control group showed no significant difference for rate of chemotherapy disruption [10.3% vs. 25.9%, pooled OR = 0.42, 95%CI (0.11, 1.58), P = 0.20] (Table 3), overall mortality [1.1% vs. 3.8%, pooled OR = 0.37, 95%CI (0.07, 2.04), P = 0.25] (Table 3) and mortality attributable to HBV reactivation [0% vs. 0.01%, pooled OR = 0.25, 95%CI (0.01, 6.82), P = 0.41] (Table 3). There was significant heterogeneity in the rate of chemotherapy disruption (P = 0.08, Table 3) and no significant heterogeneity in overall mortality (P = 0.99, Table 3). The difference in overall mortality still was not statistically significant (P = 0.41, Table 3) in which the study with the least sample (12) was removed. However, the rate of chemotherapy disruption was lower in the prophylactic group than in the control group by omitting the study of Long et al. (25) which was the origin of heterogeneity (P = 0.001, Table 3). Heterogeneity and sensitive analysis were not assessed in mortality related to HBV reactivation as two studies (25, 26) reported that no patients died of HBV reactivation and only one patient died in the control group in the study of Dai et al. (12) (Table 3).

**Table 3.** Meta-Analysis of the Various Outcomes

	Trials	Participants		Chi^2^, P value; I^2^	Pooled OR, (95% CI), P value	Sensitive Analysis
		P	C			Pooled OR, (95% CI), P value; Trials Omitted
**HBV Reactivation**	4	118	167	0.97, 0.81; 0%	0.09, (0.03, 0.26), < 0.0001	0.10, (0.03, 0.32), < 0.0001; Dai et al.
**Hepatitis**	4	118	167	10.03, 0.02; 70%	0.23, (0.06, 0.92), 0.04	0.14, (0.07, 0.29), < 0.00001; Long et al.
**Hepatitis Attributable to HBV Reactivation**	4	118	167	0.57, 0.75; 0%	0.10, (0.03, 0.32), < 0.0001	0.12, (0.03, 0.39), 0.0006; Dai et al.
**Severity of hepatitis**						
Mild	4	118	167	4.04, 0.26; 26%	0.90, (0.27, 3.03), 0.87	0.76, (0.18, 3.24), 0.71; Dai et al.
Moderate	4	118	167	3.32, 0.35; 10%	0.36, (0.11, 1.26), 0.11	0.25, (0.07, 0.90), 0.03; Dai et al.
Severe	4	118	167	7.26, 0.06; 59%	0.27, (0.04, 1.88), 0.19	0.14, (0.02, 0.87), 0.04; Long et al.
Moderate and Severe	4	118	167	4.59, 0.20; 35%	0.20, (0.07, 0.58), 0.003	0.23, (0.05, 0.99), 0.05; Dai et al.
**Severity of Hepatitis Attributable to HBV Reactivation**						
Mild	4	118	167	0.22, 0.64; 0%	0.16, (0.02, 1.30), 0.09	0.16, (0.02, 1.30), 0.09; Dai et al.
Moderate	4	118	167	1.62, 0.45; 0%	0.36, (0.07, 2.03), 0.25	0.41, (0.03, 5.18), 0.49; Dai et al.
Severe	4	118	167	4.76, 0.09; 58%	0.19, (0.02, 1.84), 0.15	0.06, (0.01, 0.46), 0.007; Yeo et al.
Moderate and Severe	4	118	167	1.62, 0.45; 0%	0.16, (0.05, 0.51), 0.002	0.19, (0.05, 0.69), 0.01; Dai et al.
**Chemotherapy Disruption **	3	107	158	4.94, 0.08; 59%	0.42, (0.11, 1.58), 0.20	0.23, (0.09, 0.55), 0.001; Long et al.
**Chemotherapy Disruption Attributable to HBV Reactivation**	3	107	158	0.05, 0.83; 0%	0.11, (0.02, 0.58), 0.01	0.11, (0.02, 0.58), 0.01; Long et al.
**Overall Mortality**	3	87	106	0.03, 0.99; 0%	0.37, (0.07, 2.04), 0.25	0.38, (0.04, 3.76), 0.41; Dai et al.
**Mortality Attributable to HBV Reactivation**	3	87	106	Not available	0.25, (0.01, 6.82), 0.41	Not performed

Abbreviations: CI, confidence intervals; C, the control group; OR, odds ratio; P, the prophylactic lamivudine group.

### 3.2. Second outcome

There was no significant difference between the prophylactic lamivudine and the control group in incidence of mild hepatitis [6.8% vs. 9.6%, pooled OR = 0.90, 95%CI (0.27, 3.03), P = 0.87] (Table 3), moderate hepatitis [3.4% vs. 13.2%, pooled OR = 0.36, 95%CI (0.11, 1.26), P = 0.11] (Table 3), mild hepatitis attributable to HBV reactivation [0 vs. 6.0%, pooled OR = 0.16, 95%CI (0.02, 1.30), P = 0.09] (Table 3) and moderate hepatitis attributable to HBV reactivation [0.8% vs.5.4%, pooled OR = 0.36, 95%CI (0.07, 2.03), P = 0.25] (Table 3). There was no significant heterogeneity in all four outcome measures (Table 3). Sensitive analysis showed that there was fewer incidence of moderate hepatitis in the prophylactic group than in the control group (P = 0.03, Table 3) and the difference still were not statistically significant in the remaining three outcome measures (Table 3).

Additionally, there was no significant difference between the prophylactic lamivudine group and the control group in incidence of severe hepatitis [4.2% vs. 18.6%, pooled OR = 0.27, 95%CI (0.04, 1.88), P = 0.19] (Table 3) and severe hepatitis attributable to HBV reactivation [1.7% vs. 12.0%, pooled OR = 0.19, 95%CI (0.02, 1.84), P = 0.15] (Table 3), accompanied by significant heterogeneity in both incidences of severe hepatitis and severe hepatitis related to HBV reactivation, which could be attributed to the study of Long et al. (25) (P = 0.06, Table 3) and the study of Yeo et al. (23) (P = 0.09, Table 3), respectively. Sensitive analysis showed that there was fewer incidence of severe hepatitis (P = 0.04, Table 3) and severe hepatitis related to HBV reactivation (P = 0.007, Table 3) in the prophylactic group compared to the control group. Since overt hepatitis was found in moderate hepatitis and severe hepatitis clinically, combination analysis of incidences of moderate and severe hepatitis, in parallel to combination analysis of incidences of moderate and severe hepatitis related to HBV reactivation was performed. Both of the combined incidences of moderate and severe hepatitis [7.6% vs. 31.7%, pooled OR = 0.20, 95%CI (0.07, 0.58), P = 0.003] (Table 3) and the combined incidences of moderate and severe hepatitis attributable to HBV reactivation [2.5% vs. 17.4%, pooled OR = 0.16, 95%CI (0.05, 0.51), P = 0.002] (Table 3) were lower in the prophylactic lamivudine group than in the control group. There was no significant heterogeneity in both two combined incidences (Table 3). Sensitive analysis showed that the difference was still statistically significant in the combined incidences of moderate and severe hepatitis related to HBV reactivation (P = 0.01, Table 3). Nevertheless, there was only a tendency to reduce the combined incidences of moderate and severe hepatitis (P = 0.05, Table 3) using prophylactic lamivudine.

### 3.3. Publication Bias

Funnel plots detected no obvious publication bias concerning HBV reactivation (Begg's Test: P = 1.000), hepatitis (Begg's Test: P = 1.000), hepatitis attributable to HBV reactivation (Begg's Test: P = 1.000), mild hepatitis (Begg's Test: P = 1.000), moderate hepatitis (Begg's Test: P = 1.000), severe hepatitis (Begg's Test: P = 1.000), chemotherapy disruption (Begg's Test: P =1.000), incidence of combination of moderate and severe hepatitis (Begg's Test: P = 0.308), incidence of combination of moderate and severe hepatitis attributable to HBV reactivation (Begg's Test: P = 1.000). Since the low incidence of mild, moderate and severe hepatitis to HBV reactivation, chemotherapy disruption to HBV reactivation, overall mortality and mortality to HBV reactivation, the publication bias cannot be determinated by Begg's Test. All four studies reported that the antiviral agent was well tolerated and was not associated with any unexpected effects or additional toxicity.

## 4. Conclusions

Chemotherapy-induced HBV reactivation may cause varying degrees of liver damage, thus will result in disrupting chemotherapy and compromising the cancer prognosis. Prophylactic use of lamivudine could effectively prevent hepatitis B virus reactivation and reduce the incidence and severity of chemotherapy-related HBV reactivation in lymphoma patients (27, 28). However, application of prophylactic lamividine in breast cancer patients is lacking. This meta-analysis indicated that prophylactic use of lamivudine could effectively decrease the rate of HBV reactivation, incidence of hepatitis and incidence of hepatitis attributable to HBV reactivation in breast cancer patients with HBsAg positive during chemotherapy (17, 23, 26). But, incidence of mild hepatitis, whether overall or attributable to HBV reactivation, was not effectively decreased. Incidence of moderate hepatitis and incidence of severe hepatitis, whether overall or attributable to HBV reactivation, did tend to be decreased by use of prophylactic lamivudine and especially in incidence of severe hepatitis. It is possible that mild hepatitis could be effectively reverted by conventional protective liver agents in relation to moderate and severe hepatitis.

By serially monitoring HBV DNA levels and liver function (ALT), it is now recognized that, viral replication occurs 1-2 weeks before clinical hepatitis flare-up in cancer patients (24, 29, 30) and the inhibitory effect of lamivudine can be achieved after 1-week of administration (31). Initiating prophylactic administration of lamivudine at least seven days before the beginning of chemotherapy and discontinuing it at least 3–6 months after the resolution of the immuno compromised state seems reasonable. Previous studies postulated several risk factors for HBV reactivation in chemotherapy-treated patients, such as baseline serum ALT level, HBV virological marker, presence of precore mutant strain, viral genotype and HBV DNA viral load before chemotherapy (32-38). The use of anthracycline-regimens and steroids appearance also are a risk factor for HBV reactivation (17, 39). But, more patients received anthracycline in the prophylactic group than in the control group, both the rate of HBV reactivation and the incidence of hepatitis in the prophylactic group were fewer in the trial of Yeo et al. (23). Although prophylactic use of lamivudine could effectively reduce the rate of HBV reactivation, the emergence of the lamivudine-resistance is another risk factor for reactivation during prophylactic use of lamivudine (40, 41). This mainly is a result of prolonged duration of lamivudine administration (42, 43). Indeed, prolonged lamivudine therapy exceeding 6 months has been associated with an increased likelihood of treatment-emergent HBV variants with a YMDD mutation (44), which results in lamivudine resistant during continued lamivudine therapy (45, 46). The resistance may rise up to 32% after one year of treatment (47, 48). In 2004, the American Association for the Study of Liver Diseases (AASLD) recommended beginning antiviral therapyseven days before chemotherapy and continuing for six months after the completion of chemotherapy by referring to level III evidence (evidence based on clinical experience, descriptive studies, or reports of expert committees) (49). Coiffier urged the same procedures to be applied on all HBV carriers (50). In 2007, AASLD made a new suggestion that lamivudine prophylaxis for more than 6 months may be required for patients with high baseline HBV DNA (51). Newer HBV antivirals, including adefovir dipivoxil, entecavir emtricitabine and possibly clevudine, are able to suppress the replication of lamivudine-resistant HBV, as well as wildtype (47, 48, 52, 53). So, even treated with prophylactic lamivudine or after withdrawal, cancer patients who are chronic HBV infected or HBV carriers should be closely checked for serum HBV DNA levels and liver function (ALT) during and after chemotherapy (54). It was reported that restoring use of lamivudine or replacement with other anti-HBV agents could prevent HBV reactivation effectively from serum HBV DNA levels and/or ALT levels increasing (55, 56). But, delayed HBV reactivation and related-hepatic failure resulting fatality have been reported at 6-24 months after completion of chemotherapy following the withdrawal of lamivudine (57-59). Further prospective large-scale clinical trials remaining needed to establish the optimal duration for prophylactic lamivudine in breast cancer patients with HBV positive receiving chemotherapy.

The rate of chemotherapy disruption related to HBV reactivation was also significantly reduced with prophylactic lamivudine. Strikingly, a significant reduction of hepatitis related to HBV reactivation was companied with a similar reduction of chemotherapy disruption related to HBV reactivation. But the rate of chemotherapy disruption only had a tendency to decline by using prophylactic lamivudine. Larger sample trials may be clarified further. As an independent prognosis factor of breast cancer, the disruption of chemotherapy, including premature termination of chemotherapy and delay in treatment schedules, would compromise the outcome of breast cancer patients (5). Hence, reduction of chemotherapy disruption may have a positive effect on the long-term outcomes of breast cancer patients with HBsAg positive. But there are still no studies with long-term followed-up outcomes to address this issue. Although incidence of hepatitis and hepatitis related to HBV reactivation were significantly few in the prophylactic lamivudine group, overall mortality and mortality related to HBV reactivation were not significantly different between both groups. In a previous study, it was demonstrated that preemptive lamivudine therapy was superior in reducing post-chemotherapy HBV-related mortality in HBsAg+ lymphoma patients undergoing chemotherapy (15). However, another study showed that the reduction of overall mortality was not statistically different between the prophylactic lamivudine group and the control group in HBsAg positive cancer patients (17). Loomba et al. (22) synthetized quantitatively that the relative risk of preventive lamivudine for HBV-related death ranged from 0.00 to 0.20 in nine of ten studies. It does favor prophylactic use of lamivudine more than control. Zhang et al. (60) compared prophylactic use of lamivudine with treatment use with or without lamivudine in fifty-eight cancer patients with HBsAg positive during chemotherapy. The mortality in the control group was significantly higher than that of prophylactic lamivudine group (16.7% vs. 0%). In this meta-analysis, no significant differences of both overall mortality and mortality attributable to HBV reactivation may be related to the low death in the studies included. Among 4 studies in this meta-analysis, only one study was concurrent prospective random trial and the other three were not. The overall methodological quality of the included studies was relatively weak, some bias may exist. Also, all patients of these studies come from East Asia, this may be due to the reason that HBV infection is endemic in this area, and there may be a selective bias in the meta-analysis. Totally, the true benefits may not be as extreme as reported here. It is important to note that the rate of HBV reactivation; incidence of hepatitis and HBV reactivation related-hepatitis were all synthetized with random effect models even without statistical heterogeneity. Remarkably, conclusions which show that prophylactic use of lamivudine could decrease the rate of HBV reactivation, incidence of hepatitis and incidence of HBV reactivation related-hepatitis in breast cancer patients with HBsAg positive during chemotherapy are complement. Prophylactic use of lamivudine in breast cancer patients undergoing chemotherapy can reduce the rate of HBV reactivation, incidence of hepatitis and incidence of HBV reactivation related-hepatitis, with the tendency to reduce severity of hepatitis and severity of HBV reactivation related-hepatitis. Although chemotherapy disruption has only a tendency to be reduced, chemotherapy disruption related to HBV reactivation has been reduced effectively. This allows more breast cancer patients to receive adequate anti-cancer therapy, which may be interpreted as survival advantage that may become evident with long-term follow-up. Nevertheless, overall mortality and mortality related to HBV reactivation were not significantly different. The optimal duration of preventive lamivudine therapy in breast cancer patients with HBsAg positive during and after chemotherapy should be determined by further studies.
